# The Föppl–von Kármán equations of elastic plates with initial stress

**DOI:** 10.1098/rsos.220421

**Published:** 2022-05-18

**Authors:** P. Ciarletta, G. Pozzi, D. Riccobelli

**Affiliations:** MOX – Dipartimento di Matematica, Politecnico di Milano, piazza Leonardo da Vinci 32, 20133 Milano, Italy

**Keywords:** elastic plates, Föppl–von Kármán, initial stress, asymptotic theory

## Abstract

Initially, stressed plates are widely used in modern fabrication techniques, such as additive manufacturing and UV lithography, for their tunable morphology by application of external stimuli. In this work, we propose a formal asymptotic derivation of the Föppl–von Kármán equations for an elastic plate with initial stresses, using the constitutive theory of nonlinear elastic solids with initial stresses under the assumptions of incompressibility and material isotropy. Compared to existing works, our approach allows us to determine the morphological transitions of the elastic plate without prescribing the underlying target metric of the unstressed state of the elastic body. We explicitly solve the derived FvK equations in some physical problems of engineering interest, discussing how the initial stress distribution drives the emergence of spontaneous curvatures within the deformed plate. The proposed mathematical framework can be used to tailor shape on demand, with applications in several engineering fields ranging from soft robotics to four-dimensional printing.

## 1. Introduction

The Föppl–von Kármán (FvK) equations are a set of nonlinear partial differential equations describing the large deflection of linear elastic plates [1,2]. They can be derived as a formal asymptotic expansion of the three-dimensional filed theory of linear elasticity in the limit of large displacements and small strains, and associated with specific boundary conditions [3–7].

The FvK equations are notably difficult to solve, but they proved to be very useful to give theoretical insights into many physical problems where in-plane and out-of-plane unknowns can be decoupled [8–12]. The research interest in FvK equations has been recently reinvigorated by the technological possibility to fabricate shape-morphing devices using soft active materials [13–19]. These morphable plates have applications in several engineering fields, ranging from soft robotics [20,21] to the design of biomimetic structures [16]. In particular, morphological transitions can be realized by controlling the geometric frustration of a soft plate by swelling [22–24], surface accretion [25], optothermal stimuli in nematic elastomers [26] and surface tension in nano-plates [27]. For these purposes, FvK equations have been derived in cases where geometrical incompatibilities arise and the undeformed configuration of the elastic plates is no longer free of initial stresses [28].

The formal asymptotic expansion leading to the FvK model has been rigorously derived as the Γ-limit of the three-dimensional elastic problem [29,30]. This analysis has been recently extended to the case of a pre-strained plate [31–34]. The existing approaches account for the geometrical frustration using additive or multiplicative decomposition of the deformation gradient for describing the spatial distribution of residual strains given by the underlying non-Euclidean metric [35–38]. However, the FvK equations with pre-strains require the prescription of the incompatible metric of the virtual relaxed state, while in many practical cases, the distribution of residual strains remains unknown. Since a stress-free configuration cannot be physically attained by non-invasive techniques, it is more suitable to consider a more general theoretical framework where the elastic energy explicitly depends on the spatial distribution of the initial stress in the reference configurations, without the need to prescribe a stress-free state [39].

In the following, we present a formal asymptotic derivation of the FvK equations for an elastic plate with initial stresses. In §2, we introduce the constitutive theory of nonlinear elastic solids with initial stresses under the assumptions of incompressibility and material isotropy. In §3, we introduce the scaling assumption for the geometrical parameters and the initial stress components and we derive the FvK equations for an initially stressed elastic plate. In §4, we explicitly solve the derived FvK equations in some physical problems, discussing how the initial stress concentration may drive the emergence of spontaneous curvatures within the deformed plate. Concluding remarks are finally summarized in §5.

## 2. Constitutive theory for initially stressed elastic materials

Let us consider a body that occupies a simply connected domain $B_{\tau }$ in its reference configuration. We use Cartesian unit vectors (**E**_*X*_, **E**_*Y*_, **E**_*Z*_) and (**e**_*x*_, **e**_*y*_, **e**_*z*_) in the reference and spatial configurations, respectively. Let **X** = *X***E**_*X*_ + *Y***E**_*Y*_ + *Z***E**_*Z*_ be the material position vector. In this undeformed configuration, the body has an initial stress, meaning that its Cauchy stress tensor is not vanishing. We denote the initial stress tensor by τ, where $\tau \;:\;B_{\tau }\to S(R^{3})$, and $S(R^{n})$ is the set of the self-adjoint $L\;:\;R^{n}\to R^{n}$, where $L\in L(R^{n})$ is a linear application. In order to enforce the material balance of linear and angular momentum, this initial stress is such that

2.1 $$\text{Div}\tau =0,\;\tau =\tau^{T},\;\mathrm{in}\;B_{\tau },$$

where Div is the material divergence operator. We remark that if the body is residually stressed, i.e. the zero-traction boundary condition τ**N** = **0** applies to the whole boundary $\partial B_{\tau }$ with material unit normal **N**, τ must be inhomogeneous and have zero average over the volume in $B_{\tau }$ in force of the mean value theorem [40].

Let **x** = ***φ***(**X**) = *x***e**_*x*_ + *y***e**_*y*_ + *z***e**_*z*_ be the spatial material vector, so that $\varphi \;:\;B_{\tau }\to B$ be the one-to-one mapping to the deformed configuration B and F=∂φ/∂X be the deformation gradient. In the following, we deal with incompressible materials, imposing the constraint J=det F=1. We further assume that the body possesses a perfectly elastic response, defining a strain energy density Ψ per unit of reference volume as [41,42]

2.2 Ψ=Ψ(F,τ).

By standard arguments, the second Piola–Kirchhoff S and Cauchy ***σ*** stress tensors reads

2.3 $$S=\frac{\partial \Psi }{\partial F}(F,\;\tau )F^{-T}-pF^{-1}F^{-T},\;\sigma =FSF^{T},$$

where *p* is the Lagrange multiplier enforcing the incompressibility constraint. In particular, when F is equal to the identity tensor I, the Cauchy stress must be equal to the initial stress tensor τ, so that

2.4 $$\tau =\frac{\partial \Psi }{\partial F}(I,\;\tau )-p_{\tau }I,$$

where *p*_***τ***_ is the value of *p* in $B_{\tau }$. If we further assume that the elastic response is invariant after the application of a rigid-body motion, the strain energy can be expressed as a function of the three invariants of the right Cauchy–Green tensor $C=F^{T}F$, the three invariants of the initial stress tensor **τ**, plus their four mixed invariants [42,43]. Since we are interested in developing a FvK theory, we are interested in considering the minimal constitutive response that takes into account geometric nonlinearities. Accordingly, we consider in the following the constitutive response of a pre-stressed Neo-Hookean material, that is given by [44]

2.5 $$\Psi (F,\;\tau )=\frac{1}{2}(\text{tr}(\tau C)+r\;\text{tr}\;C-E),$$

where *E* is the Young modulus of the unstressed material and *r* is the real root of

2.6 $$r^{3}+I_{\tau 1}r^{2}+I_{\tau 2}r+I_{\tau 3}-{\left(\frac{E}{3}\right)}^{3}=0,$$

with $I_{\tau 1}=\text{tr}\tau$, $I_{\tau 2}=\frac{1}{2}[(I_{\tau 1}^{2}-\text{tr}(\tau^{2})]$, $I_{\tau 3}=\text{det}\;\tau$. Equation (2.6) is obtained by inverting the constitutive relation (2.3) and imposing the incompressibility constraint after a repeated application of the Cayley–Hamilton theorem. Enforcing the compatibility condition (2.4) for the initial stress, we also find that *p*_τ_ = *r*. Such an energy guarantees that the material properties are not affected by the initial stress distribution, for details see [45].

From (2.3), the constitutive equation for the initial stressed Neo-Hookean material with strain energy given by (2.5) is

2.7 $$S=rI-pC^{-1}+\tau \;\mathrm{and}\;\sigma =rB-pI+F\tau F^{T},$$

where $B=FF^{T}$ is the left Cauchy–Green tensor.

Neglecting the presence of volume bulk forces, the equilibrium conditions in the reference configuration read

2.8 $$\text{Div}\;S=0,\;S=S^{T},\;\text{in}\;B_{\tau },$$

while the zero-traction conditions at the boundary give SN=0 at $\partial B_{\tau }$. In the following, we will use these constitutive assumptions to derive a FvK theory of elastic plates with initial stress.

## 3. The Föppl–von Kármán equations for initially stressed plates

In this section, we first make the geometric assumptions of the FvK theory, using dimensional analysis to justify the asymptotic development. We later derive the constitutive equations using a variational argument, assuming different scaling for the initial stress components.

### 3.1. Geometric assumptions and asymptotic analysis

**Figure 1.**
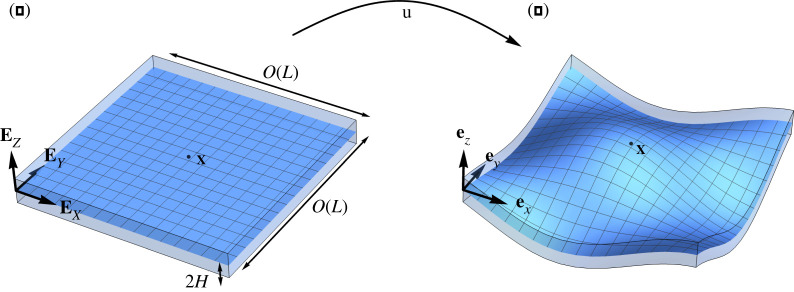
Sketch of the reference (*a*) and current (*b*) configuration of the elastic plate with initial stress. The blue surfaces represent the mid-section of the plate.

We consider an elastic plate with reference configuration $B_{\tau }=S^{m}\times [-H,H]$, where *S*^*m*^ is a closed subset in $R^{2}$, as sketched in figure 1. In particular, we require that there exists a point **P** ∈ *S*^*m*^ such that *B*_*L*_(**P**)⊆ *S*^*m*^ and *L* is much larger than the thickness 2*H* of the plate, so that *ε* = *H*/*L* ≪ 1, where $B_{L}(P)=\{Y\in R^{2}\;|\;\|Y-P\|<L\}$. The dimensionless parameter *ε* will be used to perform an asymptotic expansion. In this thin geometry, we introduce the following dimensionless variables

3.1 $$\bar{X}=\frac{X}{L},\;\bar{Y}=\frac{Y}{L},\;\bar{Z}=\frac{Z}{H},\;\bar{X}=\bar{X}E_{X}+\bar{Y}E_{Y}+\bar{Z}E_{Z}.$$

Similarly, let $\bar{\nabla }$ be the gradient operator with respect to the dimensionless variables.

Let **u** = **x** − **X** be the displacement vector describing the deformation of the plate. We denote by $u_{∥}$ the projection of **u** on the plane containing *S*^*m*^, while *w* indicates the orthogonal component of **u**. More explicitly

3.2 $$u=u_{∥}+wE_{z},\;w=u\cdot E_{Z}=H\bar{w},\;u_{∥}=Pu=u-wE_{Z}=H{\bar{u}}_{∥},$$

where $P=I-E_{Z}⊗E_{Z}$ is the projection operator, while ${\bar{u}}_{∥}$ and $\bar{w}$ are the dimensionless counterpart of $u_{∥}$ and *w*, respectively. From now on, for a generic quantity *f*, we denote by $\bar{\;f}$ its dimensionless counterpart.

We assume the classical Kirchhoff hypothesis for the in-plane displacement $u_{∥}$

3.3 $$\begin{array}{rl} & u_{∥}=H{\bar{u}}_{∥}=U^{m}-Z\nabla_{∥}w\\ \mathrm{and}\;\;\;\;\;\;\; & {\bar{u}}_{∥}={\bar{U}}^{m}-\epsilon \bar{Z}{\bar{\nabla }}_{∥}\bar{w}, \end{array}\}$$

where $U^{m}(X_{∥})=H{\bar{U}}^{m}({\bar{X}}_{∥})=u_{∥}(PX)$ represents the in-plane displacement of the point $X_{∥}$ of the middle surface, obtained through the projection of **X** on *S*^*m*^, while $\nabla_{∥}$ is the gradient operator with respect to $X_{∥}$, namely in Cartesian components $\nabla_{∥}=(\partial /\partial X,\;\partial /\partial Y,0)$, while $\nabla_{⊥}=(0,0,\partial /\partial Z)$. From the non-dimensionalization proposed in (3.1), we get

3.4 $$\nabla_{∥}=\frac{1}{L}{\bar{\nabla }}_{∥}\;\mathrm{and}\;\nabla_{⊥}=\frac{1}{H}{\bar{\nabla }}_{⊥}.$$

The out-of-plane component of the displacement vector reads

3.5 $$\begin{array}{rl} & w=H\bar{w}=\xi^{m}+\epsilon^{2}W\\ \mathrm{and}\;\;\;\;\;\;\;\; & \bar{w}={\bar{\xi }}^{m}+\epsilon^{2}\bar{W}, \end{array}\}$$

where $\xi^{m}(X_{∥})=H{\bar{\xi }}^{m}(X_{∥})=w(PX)$ is the out-of-plane displacement of the point $X_{∥}$ of the middle surface, obtained through the projection of **X** on *S*^*m*^ and $W\;:\;B_{\tau }\to R^{3}$. The field *ξ* governs the out-of-plane deformation up to an order *O*(*ε*^2^).

The assumption made in (3.3) and (3.5) imposes that the displacement is a pure bending deformation in the *Z*-direction, so that sections that are initially perpendicular to the middle surface undergo a rotation that is driven by the local curvature of the deformed middle surface. For an initially stressed plate, the validity of this scaling requires a further constraint on the scaling of the initial stress tensor, that will be discussed later.

We further assume that the middle surface components can be expanded in powers of *ε* as follows:

3.6 $$U^{m}=\sum_{n=1}^{\infty }\epsilon^{n}U_{n}^{m}=H\sum_{n=1}^{\infty }\epsilon^{n}{\bar{U}}_{n}^{m}\;\mathrm{and}\;\xi^{m}=\sum_{n=0}^{\infty }\epsilon^{n}\xi_{n}^{m}=H\sum_{n=0}^{\infty }\epsilon^{n}{\bar{\xi }}_{n}^{m}.$$

Using (3.4), the deformation gradient can be written as

3.7 $$F=I+\nabla u=I+\nabla_{∥}u+\nabla_{⊥}u=I+\epsilon {\bar{\nabla }}_{∥}\bar{u}+{\bar{\nabla }}_{⊥}\bar{u},$$

since $\bar{u}={\bar{u}}_{∥}+\bar{w}E_{Z}$, substituting (3.3) and (3.5), we get

3.8 $$\bar{u}={\bar{U}}^{m}+{\bar{\xi }}^{m}E_{Z}-\epsilon \bar{Z}{\bar{\nabla }}_{∥}{\bar{\xi }}^{m}+\epsilon^{2}\bar{W}E_{Z}+o(\epsilon^{2}).$$

We can compute ${\bar{\nabla }}_{∥}\bar{u}$ and ${\bar{\nabla }}_{⊥}\bar{u}$, obtaining

3.9 $$\begin{array}{cc} & {\bar{\nabla }}_{∥}\bar{u}={\bar{\nabla }}_{∥}{\bar{U}}^{m}+E_{Z}⊗{\bar{\nabla }}_{∥}{\bar{\xi }}^{m}-\epsilon \bar{Z}{\bar{\nabla }}_{∥}{\bar{\nabla }}_{∥}{\bar{\xi }}^{m}+o(\epsilon )\\ \mathrm{and}\;\;\;\;\; & {\bar{\nabla }}_{⊥}\bar{u}=-\epsilon {\bar{\nabla }}_{∥}{\bar{\xi }}^{m}⊗E_{Z}+\epsilon^{2}{\bar{W}}_{,\bar{Z}}E_{Z}⊗E_{Z}+o(\epsilon^{2}), \end{array}\}$$

where comma denotes the partial derivative. Thus, the right Cauchy–Green strain tensor becomes

3.10 $$\begin{array}{rl} C & =F^{T}F\\ & ={\left(I+\epsilon {\bar{\nabla }}_{∥}\bar{u}+{\bar{\nabla }}_{⊥}\bar{u}\right)}^{T}(I+\epsilon {\bar{\nabla }}_{∥}\bar{u}+{\bar{\nabla }}_{⊥}\bar{u})\\ & =I+\epsilon {\bar{\nabla }}_{∥}\bar{u}+{\bar{\nabla }}_{⊥}\bar{u}+\epsilon {\left({\bar{\nabla }}_{∥}\bar{u}\right)}^{T}+\epsilon^{2}{\left({\bar{\nabla }}_{∥}\bar{u}\right)}^{T}{\bar{\nabla }}_{∥}\bar{u}+\epsilon {\left({\bar{\nabla }}_{∥}\bar{u}\right)}^{T}{\bar{\nabla }}_{⊥}\bar{u}\\ & \;+{\left({\bar{\nabla }}_{⊥}\bar{u}\right)}^{T}+\epsilon {\left({\bar{\nabla }}_{⊥}\bar{u}\right)}^{T}{\bar{\nabla }}_{∥}\bar{u}+{\left({\bar{\nabla }}_{⊥}\bar{u}\right)}^{T}{\bar{\nabla }}_{⊥}\bar{u}\\ & =I+\epsilon^{2}A+o{\left(\epsilon \right)}^{2}, \end{array}$$

where the tensor A is defined as

3.11 $$A=2\;\text{sym}\bar{\nabla }\bar{U}-2\bar{Z}\bar{\nabla }\bar{\nabla }\bar{\xi }+(\|\bar{\nabla }\bar{\xi }{\|}^{2}+2{\bar{W}}_{,\bar{Z}})E_{Z}⊗E_{Z}+\bar{\nabla }\bar{\xi }⊗\bar{\nabla }\bar{\xi },$$

here we have set $\bar{U}={\bar{U}}_{1}^{m}$, $\bar{\xi }={\bar{\xi }}_{0}^{m}$ for the sake of simplicity. At the leading order, the incompressibility constraint reads tr A=0, that imposes

3.12 $${\bar{W}}_{,\bar{Z}}=\bar{Z}\bar{\Delta }\xi -\bar{\nabla }\cdot \bar{U}-\|\bar{\nabla }\xi {\|}^{2},$$

where $\bar{\Delta }=\bar{\nabla }\cdot \bar{\nabla }$ is the Laplace operator. The tensor A affinely depends on $\bar{Z}$. More explicitly, we write

3.13 $$A=C_{0}+\bar{Z}C_{1},$$

with

3.14 $$\begin{array}{rl} & C_{0}=2\;\text{sym}\;\bar{\nabla }\bar{U}-(\|\bar{\nabla }\bar{\xi }{\|}^{2}+2\bar{\nabla }\cdot \bar{U})E_{Z}⊗E_{Z}+\bar{\nabla }\bar{\xi }⊗\bar{\nabla }\bar{\xi }\\ \mathrm{and}\;\;\;\;\; & C_{1}=2\;\bar{\Delta }\bar{\xi }E_{Z}⊗E_{Z}-2\bar{\nabla }\bar{\nabla }\bar{\xi }. \end{array}\}$$

In appendix A, we report the explicit expression of the tensors in (3.14) using the canonical basis (**E**_*X*_, **E**_*Y*_, **E**_*Z*_). Finally, for future convenience, we also recall that the asymptotic expansion of the inverse of C reads

3.15 $$C^{-1}=I-\epsilon^{2}A+o(\epsilon^{2}).$$

### 3.2. Scaling assumptions on the initial stress

Under the constitutive assumption given by (3.5), we define the dimensionless initial stress tensor as $\bar{\tau }=\tau /E$. We assume the following scaling for the initial stress tensor:

3.16 $$\bar{\tau }=\epsilon^{2}{\bar{\tau }}_{2}+\epsilon^{3}{\bar{\tau }}_{3}+\epsilon^{4}{\bar{\tau }}_{4},$$

with

3.17 $$\begin{array}{rl} & {\bar{\tau }}_{2}={\bar{\tau }}_{XX}E_{X}⊗E_{X}+{\bar{\tau }}_{XY}(E_{X}⊗E_{Y}+E_{Y}⊗E_{X})+{\bar{\tau }}_{YY}E_{Y}⊗E_{Y},\\ & {\bar{\tau }}_{3}=2\;\text{sym}\;(({\bar{\tau }}_{XZ}E_{X}+{\bar{\tau }}_{YZ}E_{Y})⊗E_{Z})\\ \mathrm{and}\;\;\;\;\; & {\bar{\tau }}_{4}={\bar{\tau }}_{ZZ}E_{Z}⊗E_{Z}, \end{array}\}$$

where ${\bar{\tau }}_{ij}={\bar{\tau }}_{ij}(X)=O(1)$, with *i*, *j* = *X*, *Y*, *Z*. Substituting (3.16) into (2.1), the equilibrium equation in the undeformed configuration reads

3.18 $$\begin{array}{cc} & {\bar{\tau }}_{XX,\bar{X}}+{\bar{\tau }}_{XY,\bar{Y}}+{\bar{\tau }}_{XZ,\bar{Z}}=0,\\ & {\bar{\tau }}_{XY,\bar{X}}+{\bar{\tau }}_{YY,\bar{Y}}+{\bar{\tau }}_{YZ,\bar{Z}}=0\;\;\\ \mathrm{and}\;\; & {\bar{\tau }}_{XZ,\bar{X}}+{\bar{\tau }}_{YZ,\bar{Y}}+{\bar{\tau }}_{ZZ,\bar{Z}}=0. \end{array}\}$$

If the top and bottom surfaces are free of traction, i.e. ${\bar{\tau }}_{iZ}(\bar{X},\bar{Y},\pm 1)=0$, the previous equation can be further simplified by integration in the *Z*-direction

3.19 $$\begin{array}{cc} & \int_{-1}^{1}({\bar{\tau }}_{XX,\bar{X}}+{\bar{\tau }}_{XY,\bar{Y}})\;d\bar{Z}=0,\\ & \int_{-1}^{1}({\bar{\tau }}_{XY,\bar{X}}+{\bar{\tau }}_{YY,\bar{Y}})\;d\bar{Z}=0\\ \mathrm{and}\;\;\;\;\;\;\; & \int_{-1}^{1}({\bar{\tau }}_{XZ,\bar{X}}+{\bar{\tau }}_{YZ,\bar{Y}})\;d\bar{Z}=0. \end{array}\}$$

We define the averaged planar initial stress tensor ${\bar{\tau }}_{m}$ as

3.20 $${\bar{\tau }}_{m}=\frac{1}{2}\int_{-1}^{1}[{\bar{\tau }}_{XX}E_{X}⊗E_{X}+{\bar{\tau }}_{XY}(E_{X}⊗E_{Y}+E_{Y}⊗E_{X})+{\bar{\tau }}_{YY}E_{Y}⊗E_{Y}]\;d\bar{Z}.$$

The leading order of (3.19) can be automatically fulfilled by introducing the dimensionless initial Airy stress function ${\bar{\chi }}_{0}\;:\;{\bar{S}}^{m}\to R$, so that

3.21 $${\bar{\tau }}_{m}=\text{cof}({\bar{\nabla }}_{m}{\bar{\nabla }}_{m}{\bar{\chi }}_{0}),$$

where ${\bar{\nabla }}_{m}$ is the dimensionless gradient operator in $R^{2}$, cofA is the cofactor of the tensor A. For the sake of clarity, using Cartesian coordinates, we get

3.22 $${\bar{\tau }}_{m}=\left[\begin{array}{cc} {\bar{\chi }}_{0,\bar{Y}\bar{Y}} & -{\bar{\chi }}_{0,\bar{X}\bar{Y}}\\ -{\bar{\chi }}_{0,\bar{X}\bar{Y}} & {\bar{\chi }}_{0,\bar{X}\bar{X}} \end{array}\right].$$

Under these assumptions, we get *I*_***τ***_1__/*E* = *O*(*ε*^2^), *I*_***τ***_2__/*E*^2^ = *O*(*ε*^4^), *I*_***τ***_3__/*E*^3^ = *O*(*ε*^8^). From (2.6), the leading order expression of the dimensionless term $\bar{r}=r/E$ is

3.23 $$\bar{r}=\frac{1}{3}(1-\epsilon^{2}\;\text{tr}\;{\bar{\tau }}_{2})+o(\epsilon^{2}).$$

Using (2.7), (3.15) and (3.16), the components of the dimensionless second Piola–Kirchhoff stress tensor $\bar{S}=S/E$ scales as follows:

3.24 $$E_{\alpha }\cdot \bar{S}E_{\beta }=O(\epsilon^{2}),\;E_{\alpha }\cdot \bar{S}E_{Z}=O(\epsilon^{3}),\;E_{Z}\cdot \bar{S}E_{Z}=O(\epsilon^{4}),\;\alpha ,\;\beta \in \{X,\;Y\},$$

where the latter scaling is enforced by imposing that the dimensionless Lagrange multiplier $\bar{\;p}=p/E$ reads

3.25 $$\bar{\;p}=\bar{r}+\frac{\epsilon^{2}}{3}E_{Z}\cdot AE_{Z}+o(\epsilon^{2}).$$

In particular, we remark that (3.24) corresponds to the classical FvK scaling for the stress tensor, and justifies the mathematical soundness of the Kirchhoff assumption for the displacement field in (3.3) and (3.5) in the presence of initial stress components which scale as in (3.16). The leading-order components of the stress tensor read

3.26 $$\bar{S}=({\bar{S}}_{0}+\bar{Z}{\bar{S}}_{1}+\tau_{2})\epsilon^{2}+o(\epsilon^{2}),$$

with

3.27 $${\bar{S}}_{j}=\frac{1}{3}(C_{j}-E_{Z}\cdot C_{j}E_{Z}I),\;j=0,\;1.$$

We remark that both ${\bar{S}}_{0}$ and ${\bar{S}}_{1}$ are independent of $\bar{Z}$, since they depend on ${\bar{X}}_{∥}$ through $\bar{\xi }$ and $\bar{U}$.

### 3.3. Variational formulation

Let $\bar{E}=E/(EHL^{2})$ be the dimensionless counterpart of the elastic energy. From now on, we will refer only to dimensionless variables (unless differently specified) and we drop the use of the superposed line to indicate the non-dimensional quantities. We now perform a variational derivation of the generalized FvK theory for an initially stressed material by imposing the stationary conditions for the total elastic energy functional E. Neglecting traction loads at the boundary and body forces for the sake of simplicity, the first variation of the dimensionless energy reads

3.28 $$\delta E=\int_{S^{m}}\int_{Z=-1}^{1}\frac{1}{2}S\;:\;\delta C\;dZ\;dS,$$

where d*S* = d*X*d*Y* and $A\;:\;B=\text{tr}(A^{T}B)$. From (3.10), (3.13) and (3.14), the increment of the right Cauchy–Green tensor C reads

3.29 $$\delta C=(\text{sym}\nabla \delta U-2Z\nabla \nabla \delta \xi +\nabla \delta \xi ⊗\nabla \xi +\nabla \xi ⊗\nabla \delta \xi )\;\epsilon^{2}+\delta C_{ZZ}E_{Z}⊗E_{Z}+o(\epsilon^{2}),$$

where *δC*_*ZZ*_ is the increment of $E_{Z}\cdot CE_{Z}$.

Since $S_{0}$ and $S_{1}$ are symmetric and $S_{0}E_{Z}=S_{1}E_{Z}=0$, we can introduce the tensors $S_{m0}$ and $S_{m1}$ which are their projection onto $S(R^{2})$. Substituting (3.26) and (3.29) into (3.28), we get

3.30 $$\delta E=\delta E_{s}+\delta E_{b},$$

where $\delta E_{s}$ and $\delta E_{b}$ are the increments due to average planar stretching and bending of the plate, respectively. They are defined as

3.31 $$\delta E_{s}=2\epsilon^{4}\int_{S^{m}}(S_{m0}+\tau_{m})\;:\;\nabla_{m}\delta U\;dS+o(\epsilon^{4})$$

and

3.32 $$\delta E_{b}=2\epsilon^{4}\int_{S^{m}}\left[-\left(\frac{1}{3}S_{m1}+M\right)\;:\;(\nabla_{m}\nabla_{m}\delta \xi )+(S_{m0}+\tau_{m})\nabla_{m}\xi \cdot \nabla_{m}\delta \xi \right]dS+o(\epsilon^{4}),$$

where M is the dimensionless tensor representing the average bending torques imposed by the initial stress, defined as

3.33 $$M=\frac{1}{2}\int_{-1}^{1}Z[\tau_{XX}E_{X}⊗E_{X}+\tau_{XY}(E_{X}⊗E_{Y}+E_{Y}⊗E_{X})+\tau_{YY}E_{Y}⊗E_{Y}]\;dZ,$$

which vanishes if *τ*_*αβ*_ are even functions of *Z*, with *α*, *β* ∈ {*X*, *Y*}.

In the following, we detail such contributions, deriving the corresponding FvK equations as the necessary conditions for their extremal values.

#### 3.3.1. Föppl–von Kármán equation for the average planar stretch

Integrating by parts (3.31) and neglecting the remainder, we get

3.34 $$\delta E_{s}=-2\epsilon^{4}\int_{S^{m}}[\nabla_{m}\cdot (S_{m0}+\tau_{m})]\cdot \delta U\;dS+b.t.,$$

where we write + b.t. to indicate boundary terms. Accordingly, imposing $\delta E_{s}=0$ for each admissible variation *δ***U**, we get $\nabla_{m}\cdot (S_{m0}+\tau_{m})=0$. This equation is automatically fulfilled by introducing the Airy stress function *χ* as:^1^

3.35 $$S_{m0}+\tau_{m}=\text{cof}\nabla_{m}\nabla_{m}\chi ,$$

Substituting (3.21)–(3.27) into (3.35), we finally obtain

3.36 $$\Delta_{m}^{2}(\chi -\chi_{0})+[\xi ,\;\xi ]=\Delta_{m}^{2}\chi +([\xi ,\;\xi ]-C_{G})=0,$$

where $\Delta_{m}=\nabla_{m}\cdot \nabla_{m}$ is the Laplace operator in $R^{2}$ and the bracket operator is the Monge–Ampére bilinear form

3.37 $$[a,\;b]=\frac{1}{2}(\text{cof}\nabla_{m}\nabla_{m}a)\;:\;\nabla_{m}\nabla_{m}b$$

and $C_{G}=\Delta_{m}^{2}\chi_{0}$ plays the role of a spontaneous Gaussian curvature imposed by the planar initial stress.

#### 3.3.2. Föppl–von Kármán equation for the average bending stretch

Using (3.27) in (3.32) and neglecting the remainder, we integrate by parts, obtaining

3.38 $$\delta E_{b}=2\epsilon^{4}\int_{S^{m}}\left[\frac{4}{9}\Delta_{m}^{2}\xi -\nabla_{m}\cdot (\nabla_{m}\cdot M)-\nabla_{m}\cdot ((S_{m0}+\tau_{m})\nabla_{m}\xi )\right]\delta \xi \;dS\;+b.t.$$

By substituting (3.35) into (3.38) and imposing $\delta E_{b}=0$ for each admissible variation *δξ*, we finally get

3.39 $$\frac{2}{9}\Delta_{m}^{2}\xi -\frac{1}{2}\nabla_{m}\cdot \left(\nabla_{m}\cdot M\right)-[\chi ,\xi ]=\frac{2}{9}\left(\Delta_{m}^{2}\xi -\Delta_{m}C_{M}\right)-[\chi ,\xi ]=0,$$

where $\Delta_{m}C_{M}=\frac{9}{4}\nabla_{m}\cdot (\nabla_{m}\cdot M)$, so that *C*_*M*_ represents the spontaneous mean curvature imposed by the initial stress distribution. In appendix A, we show the explicit expressions of the FvK equations in Cartesian and polar coordinates.

## 4. Physical examples

In this section, we provide a few physical examples showing the morphology of the solutions of the FvK equations (3.36)–(3.39) for different distributions of the initial stress within the elastic plate.

### 4.1. Planar initial stress

Let us start by considering a planar distribution of the initial stress. Since ***τ*** does not depend on *Z*, the corresponding average bending torques vanish, i.e. M=0. Accordingly, such an initial stress distribution may impose only a non-zero spontaneous Gaussian curvature *C*_*G*_ in (3.36), while *C*_*M*_ = 0 in (3.39). For the sake of clarity, we discuss in the following some physical examples exhibiting different spontaneous Gaussian curvatures.

#### 4.1.1. Positive spontaneous Gaussian curvature

Let R, Θ be the polar coordinates of the generic point $X_{∥}\in S^{m}$. Let us first consider an initial stress distribution having an Airy stress function given by

4.1 $$\chi_{0}(R,\;\Theta )=\frac{c^{2}}{64}R^{4},$$

with *c* being a characteristic dimensionless stress parameter, and the planar initial stress tensor ***τ***_*m*_ reads

4.2 $$\tau_{m}=\frac{c^{2}R^{2}}{16}(E_{R}⊗E_{R}+3E_{\Theta }⊗E_{\Theta }).$$

From equation (3.36), the spontaneous Gaussian curvature is positive and constant, being

4.3 $$C_{G}=c^{2}>0.$$

We look for a solution such that the second Piola–Kirchhoff stress tensor vanishes. In such a case, equations (3.36)–(3.39) simplify as

4.4 $$[\xi ,\xi ]-c^{2}=0,\;\Delta_{m}^{2}\xi =0,$$

where the former is a Monge–Ampére equation. Using polar coordinates, we get

4.5 $$\begin{array}{rl} & [\xi ,\xi ]=-\frac{\xi_{,\Theta }^{2}}{R^{4}}+\frac{2\xi_{,R\Theta }\xi_{,\Theta }}{R^{3}}+\frac{\xi_{,\Theta \Theta }\xi_{,RR}}{R^{2}}-\frac{\xi_{,R\Theta }^{2}}{R^{2}}+\frac{\xi_{,R}\xi_{,RR}}{R}\\ \mathrm{and}\;\;\; & \Delta_{m}^{2}\xi =\frac{R^{2}\xi_{,RRRR}+2R\xi_{,RRR}-\xi_{,RR}+2\xi_{,RR\Theta \Theta }}{R^{2}}+\frac{\xi_{,R}-2\xi_{,R\Theta \Theta }}{R^{3}}+\frac{4\xi_{,\Theta \Theta }+\xi_{,\Theta \Theta \Theta \Theta }}{R^{4}}. \end{array}\}$$

A solution to equation (4.4) such that *ξ* depends only on *R* is given by

**Figure 2.**
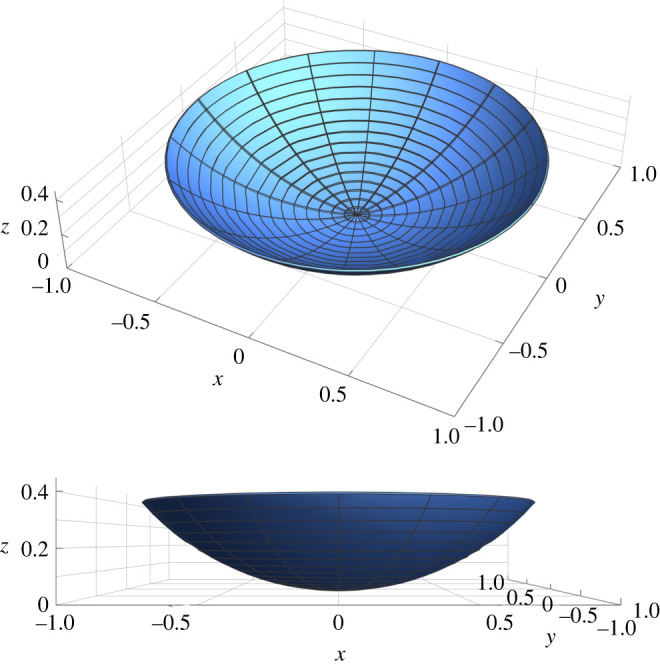
Plot of actual buckled configuration of the elastic plate (using dimensional variables) as given by (4.6) for the initial stress distribution in (4.2). We have set *H* = 0.01, *L* = 1 and *c* = 80, where $S^{m}=\{Y\in R^{2}\;|\;\|Y\|<1\}$.

4.6 $$\xi (R)=\frac{c}{2}R^{2},$$

which corresponds to a buckling of the plate, as illustrated in figure 2.

#### 4.1.2. Negative spontaneous Gaussian curvature

We now consider the following Airy stress function

4.7 $$\chi_{0}(R,\;\Theta )=-\frac{c^{2}R^{2n+4}}{{\left(4n^{2}+12n+8\right)}^{2}},\;n\in N,$$

with *c* being a characteristic dimensionless stress parameter. Accordingly, the planar initial stress reads

4.8 $$\tau_{m}=-\frac{c^{2}(2n+4)R^{2n+2}}{{\left(4n^{2}+12n+8\right)}^{2}}(E_{R}⊗E_{R}+(3+2n)E_{\Theta }⊗E_{\Theta }).$$

From (3.36), the spontaneous Gaussian curvature is negative, being

4.9 $$C_{G}=-c^{2}R^{2n}<0,$$

where we have a constant Gaussian curvature if *n* = 0. Seeking for a solution that cancels the second Piola–Kirchhoff stress tensor, equations (3.36)–(3.39) become a Monge–Ampére and a biharmonic equation, respectively, namely

4.10 $$[\xi ,\xi ]+c^{2}R^{2n}=0\;\mathrm{and}\;\Delta_{m}^{2}\xi =0.$$

It is easy to check that these equations admit a solution for each n∈N of the form

4.11 $$\xi (R,\;\Theta )=\frac{cR^{n+2}\sin((n+2)\Theta )}{n^{2}+3n+2}.$$

**Figure 3.**
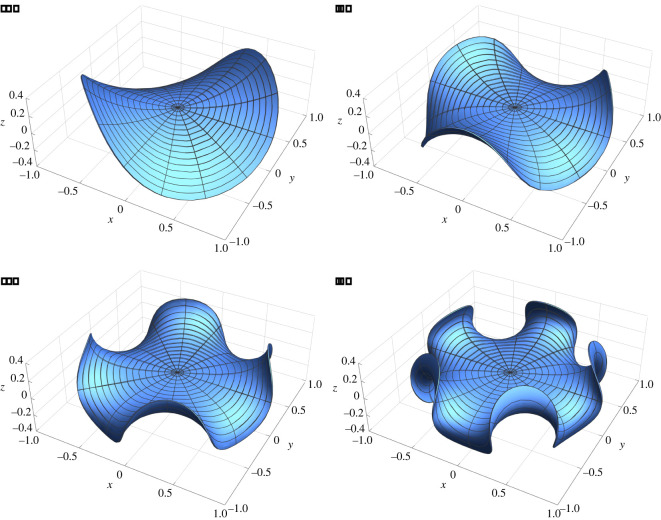
Plot of actual buckled configuration of the elastic plate (using dimensional variables) as given by (4.11) for the initial stress distribution in (4.8). We have set *H* = 0.01 and *L* = 1, where $S^{m}=\{Y\in R^{2}\;|\;\|Y\|<1\}$. The other parameters are (*a*) *n* = 0, *c* = 80; (*b*), *n* = 1, *c* = 200; (*c*), *n* = 2, *c* = 400; (*d*), *n* = 3, *c* = 800.

In particular, the solution for *n* = 0 corresponds to a twisting of the plate, as illustrated in figure 3.

#### 4.1.3. Concentrated Gaussian curvature in thin plates

In this subsection, we consider the case of large deflections *ξ*, so that the bending term $\Delta_{m}^{2}\xi$ in equation (3.39) can be neglected [36]. In this case, if *χ* = 0 the second FvK equation is automatically satisfied while the first FvK equation (3.36) becomes the Monge–Ampére equation

4.12 $$[\xi ,\xi ]=\Delta_{m}^{2}\chi_{0}.$$

We now consider the initial Airy stress function

4.13 $$\chi_{0}(R,\;\Theta )=\frac{\alpha R^{2}}{4}(\log R-1),$$

which corresponds to the planar initial stress tensor

4.14 $$\tau_{m}=\frac{\alpha }{4}(2\log R+1)I-\frac{\alpha }{2}E_{R}⊗E_{R}.$$

We get that $\Delta_{m}^{2}\chi_{0}=\alpha \delta (R)/R$, where *δ*(*R*) is a Dirac delta such that

4.15 $$\int_{0}^{+\infty }f\;(R)\delta (R)\;dR=f\;(0).$$

If the second Piola–Kirchhoff stress tensor vanishes, the first FvK equation (3.36) becomes

4.16 $$-\frac{\xi_{,\Theta }^{2}}{R^{4}}+\frac{2\xi_{,R\Theta }\xi_{,\Theta }}{R^{3}}+\frac{\xi_{,\Theta \Theta }\xi_{,RR}}{R^{2}}-\frac{\xi_{,R\Theta }^{2}}{R^{2}}+\frac{\xi_{,R}\xi_{,RR}}{R}=\alpha \frac{\delta (R)}{R},$$

which admits as solutions the functions ξ(R, Θ)=g(Θ)R, where g(Θ) must satisfy the relation [8]

4.17 $$\int_{0}^{2\pi }g(\Theta )(g^{\prime \prime }(\Theta )+g(\Theta ))\;d\Theta =4\pi \alpha .$$

Some solutions of equation (4.16) are given by Xu *et al*. [47]

4.18 $$\begin{array}{rl} & \xi (R,\;\Theta )=\sqrt{2\alpha }R,\;\;\;\;\text{if}\;\alpha >0\\ \mathrm{and}\;\;\;\; & \xi (R,\;\Theta )=2R\sqrt{\frac{-\alpha }{m^{2}-1}}\sin(m\theta ),\;\text{if }\alpha <0,\text{ with }m\in N\setminus \{0,\;1\}. \end{array}\}$$

**Figure 4.**
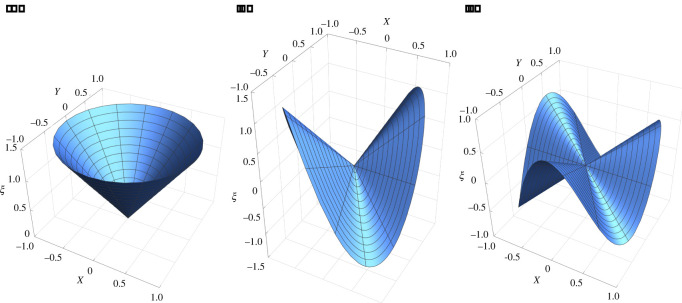
Plot of the vertical deflection *ξ* of the elastic plate as given by (4.18) for the initial stress distribution in (4.14) where we set *α* = 1 (*a*) and *α* = −1 (*b*,*c*). In the solutions for *α* = −1, we fix *m* = 2 (*b*) and *m* = 3 (*c*).

The plots of some of these solutions are shown in figure 4.

#### 4.1.4. Inhomogeneous spontaneous Gaussian curvature

Let us now consider an initial Airy stress function given by

4.19 $$\chi_{0}(X,\;Y)=-c^{2}\;e^{-2k\;X},$$

with *c* being a characteristic dimensionless stress parameter and *k* > 0 a characteristic dimensionless decay length. It corresponds to a uniaxial compression along the *Y*-axis that is inhomogeneous along the *X*-axis, and the only non vanishing initial stress component is

4.20 $$\tau_{YY}^{m}=-4k^{2}c^{2}\;e^{-2kX}.$$

From (3.36), the spontaneous Gaussian curvature is also inhomogeneous, being

4.21 $$C_{G}=-16c^{2}k^{4}\;e^{-2kX}.$$

This inhomogeneous expression for *C*_*G*_ was obtained in [28] using a different approach, based on imposing an incompatible distortion of the elastic metric. Through the cancellation of the second Piola–Kirchhoff stress tensor in the actual configuration, equations (3.36)–(3.39) simplify as the following Monge–Ampére and biharmonic equations:

4.22 $$[\xi ,\xi ]+16c^{2}k^{4}\;e^{-2kX}=0,\;\Delta_{m}^{2}\xi =0.$$

Assuming variable separation, we set *ξ* = *f*(*Y*) e^−*kX*^ so that (4.22) can be transformed in the following nonlinear ordinary differential system:

4.23 $$16c^{2}k^{2}-f^{\prime 2}+f\;f^{\prime \prime }=0\;\mathrm{and}\;k^{4}f+2k^{2}f^{\prime \prime }+f^{⁗}=0.$$

A solution of (4.23) is given by *f* = 4*c* sin (*kY*), so that the vertical deflection is

**Figure 5.**
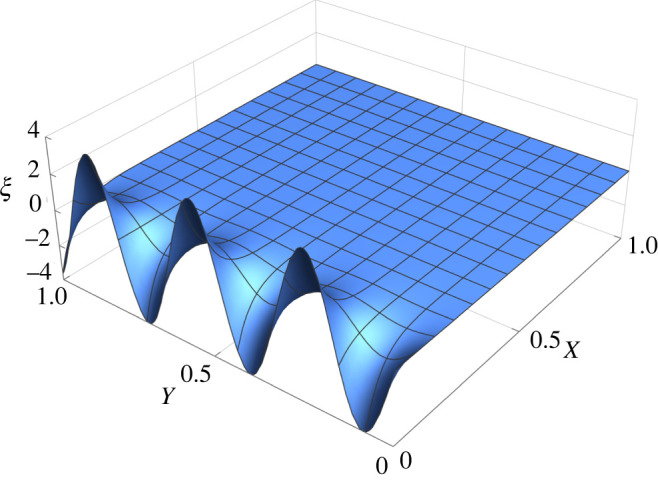
Plot of the vertical deflection *ξ* of the elastic plate as given by (4.24) for the initial stress distribution in (4.20) where we set *c* = 1 and *k* = 20.

4.24 $$\xi =4c\sin(kY)\;e^{-kX},$$

that corresponds to a sinusoidal oscillation along the *Y*-axis that decays exponentially along the *X*-axis with a decay length *k* and an amplitude proportional to *c*. The resulting morphology of the middle plane of the initially stressed plate is depicted in figure 5.

### 4.2. Initial stress varying along the plate thickness

We finally consider the case in which the initial stress is allowed to vary along the plate thickness. For the sake of simplicity, we take τ=τ(X,Z), with *τ*_*YY*_ = *τ*_*XY*_ = *τ*_*YZ*_ = 0. In this case, from (3.18), we can define an Airy stress function Φ=Φ(X,Z) such that

4.25 $$\tau_{XX}=\Phi_{,ZZ},\;\tau_{XZ}=-\Phi_{,XZ}\;\mathrm{and}\;\tau_{ZZ}=\Phi_{,XX}.$$

In particular, assuming that *τ*_*XZ*_|_*Z*±1_ = *τ*_*ZZ*_|_*Z*±1_ = 0, a leading order expansion of Φ along the vertical direction gives

4.26 $$\Phi =\left(\frac{Z^{3}}{3}-Z\right)(a_{1}X+a_{0})+b\;Z^{2},$$

where *a*_0_, *a*_1_ and *b* are dimensionless stress parameters. The only non-zero components of the average initial stress tensor $\tau_{m}$ and torque tensor M are the following:

4.27 $$\tau_{XX}^{m}=2b,\;\tau_{XZ}^{m}=\frac{2}{3}a_{1},\;M_{XX}=\frac{2}{3}(a_{1}X+a_{0}).$$

Accordingly, the parameter *b* determines the characteristic amplitude of the average initial stress, while *a*_0_, *a*_1_ determine the magnitude of the initial bending torque. Since $\tau_{XX}^{m}$ is a constant, the initial stress distribution has zero spontaneous Gaussian curvature, i.e. *C*_*G*_ = 0. Assuming *ξ* = *ξ*(*X*), since [*ξ*, *ξ*] = 0 equation (3.36) is automatically satisfied if *χ* = *χ*_0_ = *bY*^2^. The torque distribution in (4.27) imposes a spontaneous average curvature $C_{M}=\frac{3}{2}(a_{1}X+a_{0})$, and equation (3.39) reads

4.28 $$\frac{2}{9}\xi^{{\;}^{\prime \prime }}-b\xi -\frac{1}{3}(a_{1}X+a_{0})=0.$$

If the average initial stress is zero, i.e. if *b* = 0, then the solution corresponds to a bending deflection having exactly the curvature *C*_*M*_, with

4.29 $$\xi =\frac{3}{4}\left(a_{0}X^{2}-\frac{\left(a_{1}X^{3}\right)}{3}\right)+C_{1}+C_{2}X,$$

where *C*_1_, *C*_2_ are two constants of integration that must be fixed by boundary conditions.

If the average initial stress does not vanish, i.e. *b* ≠ 0, the deflection reads

4.30 $$\xi =-\frac{a_{0}+a_{1}X}{3b}+C_{1}\sinh\left(\frac{3X\sqrt{b}}{\sqrt{2}}\right)+C_{2}\cosh\left(\frac{3X\sqrt{b}}{\sqrt{2}}\right).$$

**Figure 6.**
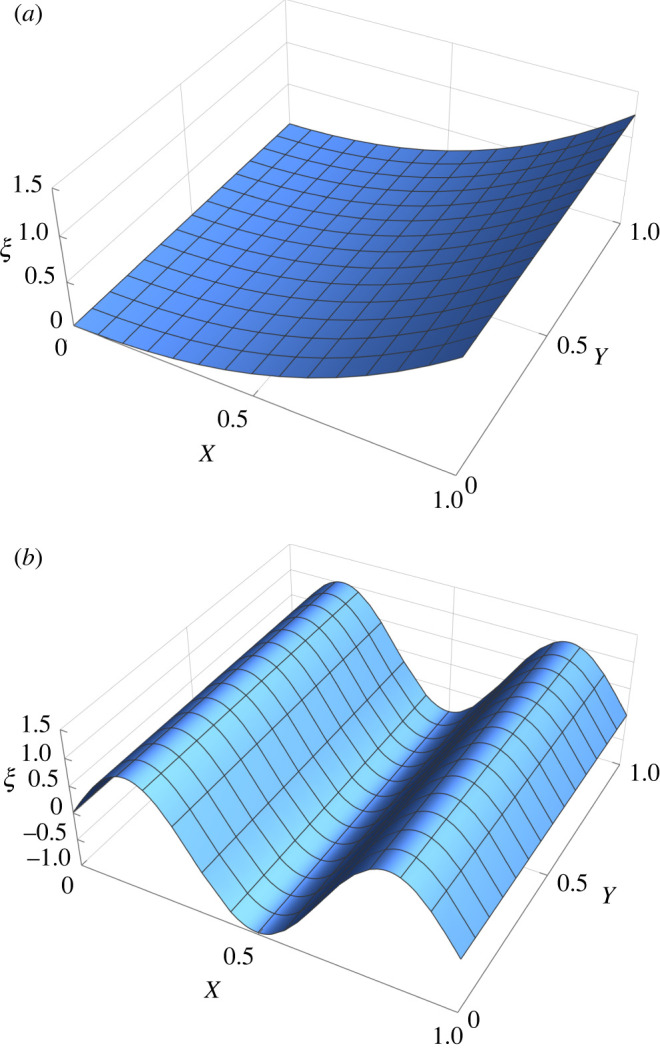
Plot of the vertical deflection *ξ* of the elastic plate, with the initial stress distribution given by (4.27) setting *a*_0_ = 1, *a*_1_ = 2. (*a*) Spontaneous bending with zero average initial stress $\tau_{XX}^{m}$, setting *b* = 0 and *C*_1_ = *C*_2_ = 0 in (4.29). (*b*) Asymmetric buckling with negative average initial stress $\tau_{XX}^{m}$, setting *b* = −20 and *C*_1_ = −*i*, *C*_2_ = 0 for the initial stress distribution in (4.30).

If the average uniaxial stress is tensile, i.e. *b* > 0, than the deflection has an exponential trend; if it is compressive, i.e. *b* < 0, than the deflection has the characteristic sinusoidal behaviour of an Euler buckling where the symmetry is broken by a spontaneous average curvature. Two possible bending morphologies of the middle line of the plate are depicted in figure 6.

Thus, in the limit of narrow plates, the governing equation (4.28) recovers the Euler beam theory for elastic rods, where the average initial stress $\tau_{XX}^{m}$ appears as a traction load, and *M*_*XX*_ as a distributed torque provoking a spontaneous curvature.

## 5. Concluding remarks

In this work, we have derived the FvK equations for an elastic plate with initial stress. The reference configuration of the plate is a parallelepiped, whose thickness 2*H* along the *z*-direction is much smaller than the characteristic length *L* of its edges. We have identified with *ε* = *H*/*L* the dimensionless parameter describing the thinness of the plate. Adopting the theoretical framework of initially stressed materials developed in [41], the elastic energy of the plate is obtained by an asymptotic expansion with respect to the small parameter *ε*.

More explicitly, we have assumed that the plate is composed of an incompressible neo-Hookean material, whose strain energy depends on both the deformation gradient and the initial stress, see (2.5). Assuming the classical Kirchhoff hypothesis for the displacement of the plate (see (3.3)–(3.5)), we used the scaling for the initial stress components reported in (3.16).

Under these assumptions, we have obtained the balance equation through a variational approach, by enforcing that the energy functional be stationary. The equilibrium equations (3.36) and (3.39) generalize the FvK equations for the average planar and bending stretch, respectively, to the case of initially stressed plates.

We have solved the FvK equations in some physical examples of engineering interest. By tuning the initial stress distribution within the plate, we have obtained buckled configurations exhibiting a constant positive or negative Gaussian curvature. Furthermore, it is possible to obtain surfaces with non-constant Gaussian curvature by properly tuning the initial stress of the plate. In particular, we propose some new solutions with a radially inhomogeneous Gaussian curvature. We have also recovered some known explicit solutions of the FvK equations, such as the conical solutions proposed by Ben Amar & Pomeau [8] for thin plates, where the initial stress exhibits logarithmic singularities, and the solution for a geometrically frustrated FvK plate reported in [28]. Finally, we analysed the effect of an initial stress that varies along the plate thickness. The plate spontaneously bends when the initial stress along the *X*-direction is non-negative, while it exhibits a wrinkling pattern if it is compressive, similarly to Euler buckling of elastic beams. In the case of more complex initial stress distributions, e.g. exhibiting checkerboard or labyrinth patterns, it is unlikely that analytical solutions can be found and approximated solutions of the proposed equations may be found exploiting numerical techniques, such as the finite-element method.

In conclusion, we have derived the FvK equations for an initially stressed plate using a formal dimensional reduction of a nonlinear strain energy function depending explicitly on both the deformation gradient and the initial stress tensor. The main advance of the proposed approach is to unravel the effects of the initial stress distribution on the spontaneous average and Gaussian curvatures of the plate without the need to prescribe incompatible pre-strains, as required in earlier works [28,35]. In fact, unlike pre-straining, the initial stress distribution within the body can be measured by means of non-destructive techniques, such as ultrasound elastography [48] or photoelasticity [49]. Moreover, a target initial stress distribution can be physically realized using modern digital fabrication techniques, such as four-dimensional printing [18,25] and UV lithography [50]. Thus, the results of the proposed model may be used to design residually stressed objects in proximity to an elastic bifurcation [51], with the aim of fabricating shape-shifting plates able to adapt their morphology in the presence of external stimuli, such as a chemical potential [52] or an electric field [53].

## Data Availability

This article has no additional data.

## Footnotes

1 The existence of the Airy stress function can be shown by using some standard theorems of the theory of distributions (see [46], p. 59, Théoréme VI). For multiply connected domains, one could proceed following the procedure proposed in ([7], pp. 61–66).

## Appendix A. Expression of the tensor C and of the Föppl–von Kármán equations in Cartesian coordinates

In this appendix, we express the right Cauchy–Green tensor C and the FvK equations using a Cartesian coordinate system. In the following, all the quantities are dimensionless.

Let *U*(*X*, *Y*) and *V*(*X*, *Y*) be the components of **U**(*X*, *Y*) with respect to the basis **E**_*X*_ and **E**_*Y*_, namely

A 1 $$U(X,\;Y)=U(X,\;Y)E_{X}+V(X,\;Y)E_{Y}.$$

The right Cauchy–Green tensor reads $C=I+\epsilon^{2}A+o(\epsilon^{2})$, where $A=C_{0}+ZC_{1}$ and

A 2 $$\begin{array}{rl} & C_{0}=\left[\begin{array}{ccc} \xi_{,X}^{2}+2U_{,X} & \xi_{,X}\xi_{,Y}+U_{,Y}+V_{,X} & 0\\ \xi_{,X}\xi_{,Y}+U_{,Y}+V_{,X} & \xi_{,Y}^{2}+2V_{,Y} & 0\\ 0 & 0 & -\|\nabla \xi {\|}^{2}-2U_{,X}-2V_{,Y} \end{array}\right],\\ & C_{1}=\left[\begin{array}{ccc} -2\xi_{,XX} & -2\xi_{,XY} & 0\\ -2\xi_{,XY} & -2\xi_{,YY} & 0\\ 0 & 0 & 2\Delta \xi \end{array}\right]. \end{array}$$

Finally, in order to retrieve the FvK equations using the canonical vector basis (**E**_*X*_, **E**_*Y*_, **E**_*Z*_), we recall that

A 3 $$[a,b]=\frac{1}{2}(a_{,XX}b_{,YY}+a_{,YY}b_{,XX}-2a_{,XY}b_{,XY}).$$

so that equation (3.36) becomes

A 4 $$\begin{array}{rl} & \xi_{,XX}\xi_{,YY}-\xi_{,XY}^{2}+\chi_{,XXXX}+2\chi_{,XXYY}+\chi_{,YYYY}-{\chi_{0}}_{,XXXX}-2{\chi_{0}}_{,XXYY}-{\chi_{0}}_{,YYYY}=0, \end{array}$$

while equation (3.39) is given by

A 5 $$\begin{array}{rl} & \frac{2}{9}(\xi_{,XXXX}+2\xi_{,XXYY}+\xi_{,YYYY})-\frac{1}{2}(M_{XX,XX}+2M_{XY,XY}+M_{YY,YY})+\frac{1}{2}(2\xi_{,XY}\chi_{,XY}-\xi_{,XX}\chi_{,YY}-\xi_{,YY}\chi_{,XX})=0. \end{array}$$

## References

[1] Föppl A. 1921 Vorlesungen über technische Mechanik, vol. 6. Leipzig: BG Teubner.

[2] von Kármán T. 1907 Festigkeitsprobleme im Maschinenbau. In *Mechanik* (eds F Klein, C Müller), pp. 311–385. Leipzig: Springer.

[3] Goodier JN. 1938 On the problems of the beam and the plate in the theory of elasticity. Transactions of the Royal Society of Canada, 3rd Series 32, 65-88.

[4] Friedrichs K, Dressler R. 1961 A boundary-layer theory for elastic plates. Commun. Pure Appl. Math. **14**, 1-33. (10.1002/cpa.3160140102)

[5] Gol’denveizer A. 1962 Derivation of an approximate theory of bending of a plate by the method of asymptotic integration of the equations of the theory of elasticity. J. Appl. Math. Mech. **26**, 1000-1025. (10.1016/0021-8928(62)90161-2)

[6] Ciarlet PG. 1980 A justification of the von Kármán equations. Arch. Ration. Mech. Anal. **73**, 349-389. (10.1007/BF00247674)

[7] Ciarlet PG, Rabier P. 2006 Les équations de von Kármán, vol. 826. New York, NY: Springer.

[8] Ben Amar M, Pomeau Y. 1997 Crumpled paper. Proc. R. Soc. Lond. A **453**, 729-755. (10.1098/rspa.1997.0041)

[9] Cerda E, Mahadevan L. 2003 Geometry and physics of wrinkling. Phys. Rev. Lett. **90**, 074302. (10.1103/PhysRevLett.90.074302) 12633231

[10] Audoly B, Boudaoud A. 2008 Buckling of a stiff film bound to a compliant substrate–Part I:: formulation, linear stability of cylindrical patterns, secondary bifurcations. J. Mech. Phys. Solids **56**, 2401-2421. (10.1016/j.jmps.2008.03.003)

[11] Audoly B, Pomeau Y. 2010 Elasticity and Geometry: from Hair Curls to the Non-linear Response of Shells. Oxford, UK: Oxford University Press.

[12] Vella D, Davidovitch B. 2017 Indentation metrology of clamped, ultra-thin elastic sheets. Soft Matter **13**, 2264-2278. (10.1039/C6SM02451C) 28262872

[13] Fernandes A, Maurini C, Vidoli S. 2010 Multiparameter actuation for shape control of bistable composite plates. Int. J. Solids Struct. **47**, 1449-1458. (10.1016/j.ijsolstr.2010.02.007)

[14] Felton S, Tolley M, Demaine E, Rus D, Wood R. 2014 A method for building self-folding machines. Science **345**, 644-646. (10.1126/science.1252610) 25104380

[15] Zhang T, Li X, Gao H. 2014 Defects controlled wrinkling and topological design in graphene. J. Mech. Phys. Solids **67**, 2-13. (10.1016/j.jmps.2014.02.005)

[16] Gladman AS, Matsumoto EA, Nuzzo RG, Mahadevan L, Lewis JA. 2016 Biomimetic 4D printing. Nat. Mater. **15**, 413-418. (10.1038/nmat4544) 26808461

[17] Bowick MJ, Košmrlj A, Nelson DR, Sknepnek R. 2017 Non-Hookean statistical mechanics of clamped graphene ribbons. Phys. Rev. B **95**, 104109. (10.1103/PhysRevB.95.104109)

[18] van Rees WM, Matsumoto EA, Gladman AS, Lewis JA, Mahadevan L. 2018 Mechanics of biomimetic 4D printed structures. Soft Matter **14**, 8771-8779. (10.1039/C8SM00990B) 30335118

[19] Tao Y et al. 2021 Morphing pasta and beyond. Sci. Adv. **7**, eabf4098. (10.1126/sciadv.abf4098) 33952522 PMC8099191

[20] Feinberg AW, Feigel A, Shevkoplyas SS, Sheehy S, Whitesides GM, Parker KK. 2007 Muscular thin films for building actuators and powering devices. Science **317**, 1366-1370. (10.1126/science.1146885) 17823347

[21] Hines L, Petersen K, Lum GZ, Sitti M. 2017 Soft actuators for small-scale robotics. Adv. Mater. **29**, 1603483. (10.1002/adma.201603483) 28032926

[22] Kim J, Yoon J, Hayward RC. 2010 Dynamic display of biomolecular patterns through an elastic creasing instability of stimuli-responsive hydrogels. Nat. Mater. **9**, 159-164. (10.1038/nmat2606) 20023633

[23] Pezzulla M, Shillig SA, Nardinocchi P, Holmes DP. 2015 Morphing of geometric composites via residual swelling. Soft Matter **11**, 5812-5820. (10.1039/C5SM00863H) 26076671

[24] van Rees WM, Vouga E, Mahadevan L. 2017 Growth patterns for shape-shifting elastic bilayers. Proc. Natl Acad. Sci. USA **114**, 11 597-11 602. (10.1073/pnas.1709025114) 29078336 PMC5676909

[25] Zurlo G, Truskinovsky L. 2017 Printing non-Euclidean solids. Phys. Rev. Lett. **119**, 048001. (10.1103/PhysRevLett.119.048001) 29341729

[26] Mihai LA, Goriely A. 2020 A plate theory for nematic liquid crystalline solids. J. Mech. Phys. Solids **144**, 104101. (10.1016/j.jmps.2020.104101)

[27] Yue Y, Ru C, Xu K. 2017 Modified von Kármán equations for elastic nanoplates with surface tension and surface elasticity. Int. J. Non-Linear Mech. **88**, 67-73. (10.1016/j.ijnonlinmec.2016.10.013)

[28] Dervaux J, Ciarletta P, Ben Amar M. 2009 Morphogenesis of thin hyperelastic plates: a constitutive theory of biological growth in the Föppl–von Kármán limit. J. Mech. Phys. Solids **57**, 458-471. (10.1016/j.jmps.2008.11.011)

[29] Friesecke G, James RD, Müller S. 2006 A hierarchy of plate models derived from nonlinear elasticity by gamma-convergence. Arch. Ration. Mech. Anal. **180**, 183-236. (10.1007/s00205-005-0400-7)

[30] Neukamm S, Velčić I. 2013 Derivation of a homogenized von-Karman plate theory from 3D nonlinear elasticity. Math. Models Methods Appl. Sci. **23**, 2701-2748. (10.1142/S0218202513500449)

[31] Lewicka M, Mahadevan L, Pakzad MR. 2011 The Föppl-von Kármán equations for plates with incompatible strains. Proc. R. Soc. A **467**, 402-426. (10.1098/rspa.2010.0138)

[32] Bhattacharya K, Lewicka M, Schöffner M. 2016 Plates with incompatible prestrain. Arch. Ration. Mech. Anal. **221**, 143-181. (10.1007/s00205-015-0958-7)

[33] Ricciotti D, Lewicka M, Raoult A. 2017 Plates with incompatible prestrain of high order. Annales de l’Institut Henri Poincaré C, Analyse non linéaire **34**, 1883-1912. (10.1016/j.anihpc.2017.01.003)

[34] Bolaños SJ. 2020 On the dimension reduction in prestrained elasticity (survey). In Advances in Mathematical Sciences. Association for Women in Mathematics Series, vol. 21 (eds B Acu, D Danielli, M Lewicka, A Pati, RV Saraswathy, M Teboh-Ewungkem), pp. 311-324. Cham, Switzerland: Springer.

[35] Klein Y, Efrati E, Sharon E. 2007 Shaping of elastic sheets by prescription of non-Euclidean metrics. Science **315**, 1116-1120. (10.1126/science.1135994) 17322058

[36] Dervaux J, Ben Amar M. 2008 Morphogenesis of growing soft tissues. Phys. Rev. Lett. **101**, 068101. (10.1103/PhysRevLett.101.068101) 18764507

[37] Efrati E, Sharon E, Kupferman R. 2009 Elastic theory of unconstrained non-Euclidean plates. J. Mech. Phys. Solids **57**, 762-775. (10.1016/j.jmps.2008.12.004)

[38] Efrati E, Sharon E, Kupferman R. 2013 The metric description of elasticity in residually stressed soft materials. Soft Matter **9**, 8187-8197. (10.1039/c3sm50660f)

[39] Ciarletta P, Destrade M, Gower AL. 2016 On residual stresses and homeostasis: an elastic theory of functional adaptation in living matter. Sci. Rep. **6**, 1-8. (10.1038/srep24390) 27113413 PMC4845028

[40] Hoger A. 1985 On the residual stress possible in an elastic body with material symmetry. Arch. Ration. Mech. Anal. **88**, 271-289. (10.1007/BF00752113)

[41] Shams M, Destrade M, Ogden RW. 2011 Initial stresses in elastic solids: Constitutive laws and acoustoelasticity. Wave Motion **48**, 552-567. (10.1016/j.wavemoti.2011.04.004)

[42] Merodio J, Ogden RW, Rodríguez J. 2013 The influence of residual stress on finite deformation elastic response. Int. J. Non-Linear Mech. **56**, 43-49. (10.1016/j.ijnonlinmec.2013.02.010)

[43] Spencer A. 1971 Part III. Theory of invariants. In *Continuum Physics* (ed. A Eringen), vol. 1, pp. 239–353. Oxford, UK: Academic Press.

[44] Gower AL, Ciarletta P, Destrade M. 2015 Initial stress symmetry and its applications in elasticity. Proc. R. Soc. A **471**, 20150448. (10.1098/rspa.2015.0448)

[45] Riccobelli D, Agosti A, Ciarletta P. 2019 On the existence of elastic minimizers for initially stressed materials. Phil. Trans. R. Soc. A **377**, 20180074. (10.1098/rsta.2018.0074) 30879420 PMC6452035

[46] Schwartz L. 1957 Théorie des distributions à valeurs vectorielles. I. In *Annales de l’institut Fourier (Grenoble)* (ed. N Brelot), vol. 7, pp. 1–141. Chartres, France: Imprimerie Durand.

[47] Xu F, Fu C, Yang Y. 2020 Water affects morphogenesis of growing aquatic plant leaves. Phys. Rev. Lett. **124**, 038003. (10.1103/PhysRevLett.124.038003) 32031851

[48] Li GY, He Q, Mangan R, Xu G, Mo C, Luo J, Destrade M, Cao Y. 2017 Guided waves in pre-stressed hyperelastic plates and tubes: application to the ultrasound elastography of thin-walled soft materials. J. Mech. Phys. Solids **102**, 67-79. (10.1016/j.jmps.2017.02.008)

[49] Nienhaus U, Aegerter-Wilmsen T, Aegerter CM. 2009 Determination of mechanical stress distribution in Drosophila wing discs using photoelasticity. Mech. Dev. **126**, 942-949. (10.1016/j.mod.2009.09.002) 19748573

[50] Na JH, Bende NP, Bae J, Santangelo CD, Hayward RC. 2016 Grayscale gel lithography for programmed buckling of non-Euclidean hydrogel plates. Soft Matter **12**, 4985-4990. (10.1039/C6SM00714G) 27169886

[51] Jones GW, Mahadevan L. 2015 Optimal control of plates using incompatible strains. Nonlinearity **28**, 3153-3174. (10.1088/0951-7715/28/9/3153)

[52] Andrini D, Lucantonio A, Noselli G. 2021 A theoretical study on the transient morphing of linear poroelastic plates. J. Appl. Mech. **88**, 031008. (10.1115/1.4048806)

[53] De Tommasi D, Puglisi G, Saccomandi G, Zurlo G. 2010 Pull-in and wrinkling instabilities of electroactive dielectric actuators. J. Phys. D: Appl. Phys. **43**, 325501. (10.1088/0022-3727/43/32/325501)

